# Efficacy and safety of patient-controlled epidural analgesia versus patient-controlled intravenous analgesia following open hepatectomy: A single-center retrospective study

**DOI:** 10.1016/j.heliyon.2023.e23548

**Published:** 2023-12-14

**Authors:** Xue-Peng Zhang, Wan-Ting Wei, Yong Huang, Chang-Hong Miao, Xiao-Guang Zhang, Fang Du

**Affiliations:** a Department of Anesthesiology, Zhongshan Hospital, Fudan University, Shanghai, China; b Department of Anesthesiology, Minhang Hospital, Fudan University, Shanghai, China; c Department of Anesthesiology, Jinshan Hospital, Fudan University, Shanghai, China

**Keywords:** Postoperative analgesia, ERAS, Opioid, Patient-controlled analgesia, Epidural

## Abstract

**Background:**

Postoperative analgesia is an essential component of enhanced recovery after surgery following abdominal surgery. Studies comparing the effectiveness of epidural analgesia with that of other analgesic modalities after liver surgery have reported inconsistent results. Consequently, the use of epidural analgesia for open hepatectomy is controversial.

**Objective:**

The present single-center retrospective study aimed to compare the efficacy and safety of patient-controlled epidural analgesia (PCEA) and patient-controlled intravenous analgesia (PCIA) in adults undergoing open hepatectomy.

**Methods:**

Patients who underwent open hepatectomy between January 2018 to December 2019 at Zhongshan Hospital, Fudan University were retrospectively analyzed. Propensity score matching was used to adjust baseline information between the PCEA and PCIA groups. The primary outcome measure was scores of the numeric rating scales (NRSs) for resting, exercise, and nocturnal pain at postoperative 24 h (postoperative day 1 [POD1]) and 48 h (POD2). The secondary outcome indicators included postoperative nausea and vomiting (PONV), hypotension, pruritus, respiratory depression, functional activity score (FAS), effective analgesic pump compression ratio, analgesic relief rate, discontinuation of the analgesic pump, reasons for discontinuation of the analgesic pump, and patient satisfaction with postoperative analgesia.

**Results:**

The NRS scores of the PCEA group on POD1 were significantly lower than those of the PCIA group (P < 0.05). On POD2, the difference between the two groups was significant only for motion NRS scores (P < 0.05). The PCIA group had significantly more patients with lower FAS functional class than the PCEA group (P < 0.001). The effective analgesic pump compression ratio and the analgesic relief rate at 2 days after the surgery were lower in the PCEA group than in the PCIA group (P < 0.001). The incidence of pump discontinuation was higher in the PCEA group than in the PCIA group on POD2 (P = 0.044). Moreover, on POD1 and POD2, the PCEA group showed a higher incidence of pruritus and hypotension than the PCIA group (P < 0.001). Both groups showed no significant difference in PONV incidence.

**Conclusion:**

In patients undergoing open hepatectomy, PCEA was more effective than PCIA in relieving moderate to severe pain on POD1. However, improving the safety and effectiveness of PCEA remains a challenge.

## 1 Introduction

Despite recent advances in minimally invasive techniques, open hepatectomy is still a common surgical procedure. Open liver surgery is frequently associated with severe postoperative pain, and inadequate analgesia can lead to excessive stress response, increased postoperative cardiopulmonary complications, and prolonged hospital stay [1]. Postoperative analgesia is a critical component of enhanced recovery after surgery (ERAS) following abdominal surgery. Current analgesic modalities commonly used in abdominal surgery include patient-controlled intravenous analgesia (PCIA) and patient-controlled epidural analgesia (PCEA) [2].

Hepatectomy is a unique surgery wherein patients undergoing liver tumor resection may have preoperative or postoperative coagulation abnormalities that may lead to delayed epidural catheter removal or require additional fresh frozen plasma or platelet transfusion to improve coagulation [3]. Hypotension due to epidural analgesia can also lead to increased intraoperative fluid rehydration, while hypoperfusion of the liver due to hypotension can affect the recovery of residual liver function. A retrospective study that included 1153 patients showed that epidural analgesia is also an independent risk factor for postoperative acute kidney injury in hepatectomy of ≥3 liver segments [4]. The effects of postoperative epidural analgesia are also closely associated with the epidural puncture technique. Postoperative analgesia can fail because of poor positioning of the epidural catheter and its unexpected prolapse. Recent studies that compared analgesic efficacy between epidural analgesia and other analgesic modalities after liver surgery showed inconsistent results [5]. Therefore, the use of epidural analgesia in open hepatectomy remains controversial. According to the ERAS guidelines for hepatectomy published by the European ERAS Society in 2016, epidural analgesia cannot be recommended as a routine analgesic method after open hepatectomy [6]. Considering the excellent upper abdominal surgery analgesic effect of epidural anesthesia and the possibility of reducing the consumption of opioids, continuous catheter-based epidural analgesia is routinely performed in our center when patients under open hepatectomy without coagulation abnormalities. For patients under open liver resection with minor coagulation abnormalities, the anesthesiologist may also choose epidural anesthesia according to the patient's general situation.

The present single-center retrospective study compared the effectiveness and adverse effects of PCEA and PCIA during postoperative analgesia in patients undergoing open hepatectomy on the basis of postoperative complications and prognosis and determined the risk factors contributing to moderate and severe postoperative pain.

## 2 Materials and methods

This retrospective study was approved by the Ethics Committee of Zhongshan Hospital Affiliated to Fudan University (approval number: B2021-102), and the study was registered in the Chinese Clinical Trial Registry (registration number: ChiCTR2100044053). This investigation was a single-center study with data retrieved from the medical record system of Zhongshan Hospital, Fudan University. Data collection and pooling were performed by two experienced researchers. The investigators reviewed the medical records of patients undergoing surgery as defined and required in the protocol to obtain data on patient demographics, preoperative assessments, intraoperative passages, postoperative analgesic follow-up records, and postoperative regression. The hospitalization number and the date of surgery of the patients were used in combination to determine identifiers to link data from different databases, and individual data numbers were assigned to each patient. After all data coding was completed, the hospitalization number information was deleted before the data were included in the statistical analysis to secure patient-related information.

Patients who underwent open hepatectomy at Zhongshan Hospital Affiliated to Fudan University from January 1, 2018 to December 31, 2019 were enrolled in this study. Postoperative analgesia was performed by PCIA or PCEA. Patients who completed at least 2 days of postoperative analgesic follow-up were included in the data analysis. The exclusion criteria were as follows: patients with missing analgesic data due to various causes, and patients declining to provide informed consent after telephone contact. The patients were categorized into the PCEA group and the PCIA group according to the postoperative analgesia method they received following the surgery. The protocol for patients with PCEA at our institution is routinely 0.12 % ropivacaine +0.4 μg/ml sufentanil in a total volume of 250 ml, with a background dose of 3 ml/h, a bolus dose of 4 ml, and a minimum effective compression interval of 10 min. The routine protocol for PCIA is 1 μg/ml of sufentanil in a total volume of 250 ml, with a background dose of 1 ml/h, bolus dose of 3 ml and a minimum effective compression interval of 8 min. The study protocol adhered to STROBE guidelines.

The following data were collected for the present study: age, gender, body mass index (BMI), American Society of Anesthesiologists (ASA) classification, preoperative anticoagulation and antiplatelet medication use, preoperative Child's classification, Albumin-Bilirubin (ALBI) grade, surgical procedure, history of a second surgery, duration of surgery, tumor size, type of incision, requirement or no requirement for hepatic flow blockade, total duration of hepatic flow blockade, intraoperative bleeding, urine volume, intraoperative fluid replacement, postoperative hospital stay, duration of postoperative complications, and analgesic pump formulation. The primary outcome measure was scores of the numeric rating scales (NRSs) of rest, exercise, and nocturnal pain at postoperative 24 h (postoperative day 1 [POD1]) and 48 h (POD2). Analgesic follow-up was performed on POD1 and POD2 from 8 a.m. to 10 a.m. Secondary outcome indicators included postoperative nausea and vomiting (PONV), hypotension, pruritus, respiratory depression, functional activity score (FAS), effective analgesic pump compression ratio, analgesic relief rate, discontinuation of the analgesic pump, reasons for discontinuation of the pump, and patient satisfaction with postoperative analgesia. Pre- and postoperative coagulation-related tests included international normalized ratio (INR), activated partial thromboplastin time (APTT), and platelet (PLT) count. An NRS score of ≥4 was considered moderately severe pain and required further remedial management. According to the safety guidelines for epidural catheter placement and removal, INR >1.5 or APTT >40 s or PLT <80 × 10^9^/L was considered abnormal coagulation [7].

### 2.1 Statistical analysis

Continuous variables are expressed as mean ± standard deviation, and frequencies are expressed as percentages. Propensity score matching (PSM) was used to adjust baseline information between the groups and to conduct statistical analyses. Models for propensity scores included age, sex, and BMI. Patients in the PCIA and PCEA groups were matched for propensity scores at the ratio of 1:1, and residual covariate imbalance after matching was assessed by calculating standardized differences. Univariate differences between patients in the PCIA and PCEA groups were subsequently evaluated in the matched cohort by using the chi-square test or Fisher's exact test for categorical variables and the independent samples *t*-test or Wilcoxon signed-rank test for continuous variables. Hierarchical data were analyzed using the Cochrane-Armitage trend test to analyze trends in the risk associated with the two groups.

Independent samples t-tests were performed for patients' age and BMI, while the chi-square test was used for categorical variables of gender, ASA classification, Child's classification, ALBI grade, incision type, and type of surgery. The Cochran–Armitage trend test was also performed for ASA classification, Child's classification, and ALBI grade. The postoperative NRS score was used as a continuous variable for statistical analysis. NRS score ≥4 was classified as moderate or severe pain. Between-group differences in the incidence of moderate and severe pain were assumed as endpoint indicators and analyzed by the chi-square test. The variables of gender, age, and ASA classification were used as independent variables, and a binary logistic regression model was constructed to determine the influencing factors associated with the occurrence of moderate and severe pain. A chi-square test for getting or not getting out of bed was performed for both groups. A chi-square test and an additional Cochran–Armitage trend test were performed for the FAS functional scores to evaluate the effect of pain on the movement and functional status of the patients.

SPSS 26.0 and SAS 9.4 software were used for statistical analyses. A two-sided P < 0.05 was considered statistically significant.

## 3 Results

**Fig. 1:**
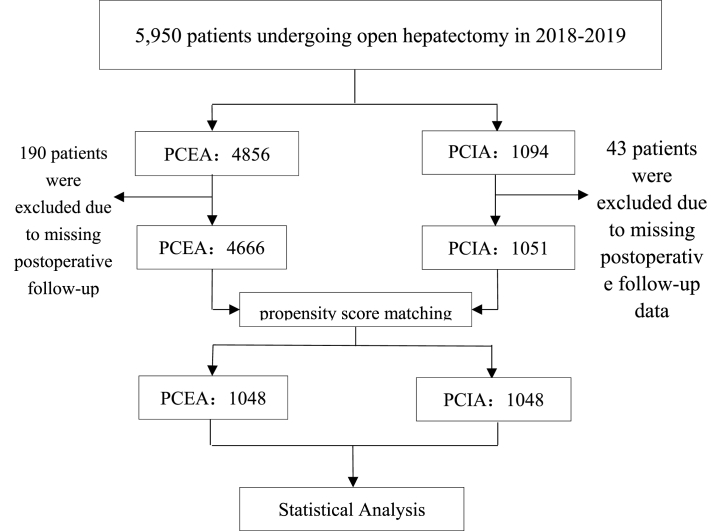
PRISMA flowchart.

A total of 5950 patients who underwent open hepatectomy during 2018–2019 were enrolled in the present study, including 4856 patients in the PCEA group and 1094 patients in the PCIA group. Of these patients, 190 patients in the PCEA group and 43 patients in the PCIA group were excluded because of missing postoperative analgesic follow-up data, and 5717 patients (4666 patients in the PCEA group and 1051 patients in the PCIA group) were finally included in the statistical analysis. The PSM method was used to match the two groups of patients with comparable baseline data. Age, sex, and BMI were incorporated into the PSM for matching, and the caliper value was set to 0.02. Finally, 1048 patients in the PECA group who could best match the PCIA group were obtained, and a new PSM database was created for further statistical analysis (See Fig. 1).

Table 1 shows the preoperative basic information and surgery-related information of the two groups after matching. No significant differences were observed in age, gender, BMI, ASA classification, preoperative serum creatinine (Scr pre), and Child's classification between the two groups. However, the two groups showed a significant difference in the type of surgery, with significantly more patients in the PCEA group who underwent simple liver tumor resection than in the PCIA group (P < 0.001). Moreover, the groups showed no significant difference in the types of incisions chosen for surgery. ALBI grade exhibited a significant difference between the two groups. The proportion of patients with ALBI grade A was significantly higher in the PCEA group than in the PCIA group; however, the proportion of patients with ALBI grade B was lower in the PCEA group than in the PCIA group (P < 0.001).

**Table 1:** Patient background and surgical characteristics.

	PCEA	PCIA	P
**Gender**			0.6439
Male	804	795	
Female	244	253	
Age	57.51（11.73）	58.46（10.83）	0.056
BMI	23.71（3.08）	23.49（3.20）	0.12
**ASA**			0.467
Ⅰ	288	309	
Ⅱ	744	718	
Ⅲ	16	21	
**Child-Pugh Score**			0.543
A	1025	1030	
B	23	17	
C	0	1	
**ALBI Grade**			<.0001
I	790	678	
II	142	259	
III	2	3	
Scr(pre)	74.99	75.71	0.6143
**Incision Type**			0.2564
Subcostal incision	975	974	
J-shaped incision	56	46	
Mercedes-bens incision	8	16	
Midline incision	6	10	
others	1	2	
**Surgery Type**			<.0001
Liver resection	986	905	
Additional organ resection	11	25	
Radiofrequency ablation	17	65	
Other surgeries	34	53	

Scr pre, preoperative serum creatinine. Data are expressed as mean (standard deviation) or number of cases (percentage).

As shown in Table 2, the analysis of the NRS scores of the two groups in the postoperative period showed that the PCEA group had significantly lower NRS scores for all three states of POD1 than the PCIA group (P < 0.05). Moreover, for POD2, the difference between the two groups was significant only for motion NRS scores, and no significant difference was observed for resting state and nocturnal NRS scores.

**Table 2:** POD1 and POD2 pain score.

		PCEA	PCIA	P
**POD1**				
	NRS（rest）	0.411（0.734）	0.688（0.951）	<.0001
	NRS（motion）	1.863（1.334）	2.527（1.417）	<.0001
	NRS（night）	0.075（0.655）	0.168（0.990）	0.012
**POD2**				
	NRS（rest）	0.161（0.441）	0.178（0.480）	0.411
	NRS（motion）	1.271（1.049）	1.500（1.048）	<.0001
	NRS（night）	0.053（0.57）	0.063（0.594）	0.679
**POD1**				
(NRS≥4)	NRS（rest）	7(0.67 %)	26(2.48 %)	0.001
	NRS（motion）	95(9.06 %)	187(17.84 %)	<.0001
	NRS（night）	14(1.35 %)	31(2.96 %)	0.01
**POD2**				
(NRS≥4)	NRS（rest）	0(0)0	1(0.01 %)	0.5
	NRS（motion）	31(2.96 %)	32(3.05 %)	0.898
	NRS（night）	12(1.15 %)	9(0.858 %)	0.511

POD1, postoperative day 1; POD2, postoperative day 2; NRS, numeric rating scales.

Data are expressed as mean (standard deviation) or number of cases (percentage).

Despite the significant differences, the mean NRS scores in all three states were less than 3 in both groups at 2 days post operation; this finding indicates that most patients had only mild pain. We further performed a comparative analysis of patients with postoperative NRS ≥4 points. The results revealed that patients in the PCIA group had a significantly higher incidence of moderate to severe pain on POD1 than patients in the PCEA group. However, on POD2, no significant difference in the incidence of moderate to severe pain was observed between the two groups.

The occurrence of moderate to severe pain (1 = yes, 0 = no) was assumed as the dependent variable, and gender, age, ASA classification, and other variables were included as independent variables to construct a binary logistic regression model. The results showed that the mode of postoperative analgesia was associated with the occurrence of moderate to severe pain at rest on POD1, with an estimated odds ratio (OR) (95 % CI) of 0.468 (0.098–0.608) (P = 0.002). Moderate to severe pain in the motor state on POD1 was associated with the mode of postoperative analgesia, with an estimated OR (95 % CI) of 0.244 (0.346–0.632) (P < 0.001).

The data presented in Table 3 reveal a significant difference in the distribution of FAS functional class between the two groups. The Cochran–Armitage trend test showed that the PCIA group had significantly more patients with lower FAS functional class than the PCEA group (P < 0.001). The FAS functional class of both groups on POD2 was A.

**Table 3:** Patient's postoperative motion status, FAS functional classification.

		PCEA	PCIA	P
FAS functional classification	**POD1**			0.0002
	A	840	767	
	B	199	259	
	C	9	22	
	**POD2**			
	A	1048	1048	
	B	0	0	
	C	0	0	

FAS, functional activity score.

The effective ratio of analgesic pump push and the analgesic relief rate in the PCEA group were significantly lower than those in the PCIA group on POD2 (P < 0.001). The two groups showed no significant difference in patients’ subjective satisfaction with the effectiveness of postoperative analgesia (Table 4).

**Table 4:** The effective ratio of analgesic pump push and the analgesic relief rate.

		PCEA	PCIA	P
Effective ratio	POD1	0.65（0.450）	0.81（0.336）	＜0.001
	POD2	0.76（0.373）	0.86（0.274）	＜0.001
Relief rate	POD1	69.08 %	86.83 %	＜0.0001
	POD2	82.73 %	92.26 %	＜0.0001

We further analyzed whether the patients discontinued the analgesic pump after surgery and the reasons for discontinuation (Table 5). The incidence of pump discontinuation was higher in the PCEA group than in the PCIA group on POD2 (P = 0.044). On POD1, the reasons for postoperative pump discontinuation differed between the two groups: hypotension and pruritus were the main reasons for pump discontinuation in the PCEA group, while PONV was the main reason for pump discontinuation in the PCIA group. The two groups showed no significant difference in the reasons for pump discontinuation on POD2.

**Table 5:** Analgesic pump discontinuation and reasons.

		PCEA（Y/N）	PCIA（Y/N）	P
Discontinuation or not				
	POD1	105/943	87/961	0.173
	POD2	36/1012	21/1027	0.044
Reasons				
	**POD1**			0.0002
	PONV	52	63	
	Pruritus	5	0	
	Hypotension	30	6	
	Others	12	13	
	**POD2**			0.231
	PONV	14	13	
	Pruritus	3	0	
	Hypotension	5	0	
	Others	11	10	

A comparison of postoperative analgesia-related adverse reactions in Table 6 showed that the incidence of pruritus and hypotension was higher in the PCEA group than in the PCIA group on both POD1 and POD2 (P < 0.001). PONV incidence was not significantly different between the two groups. The two independent-samples chi-square test was used to determine changes in the coagulation function of patients before and after surgery, and the results showed that the percentage of patients with coagulation dysfunction or oral anticoagulation and receiving antiplatelet drugs was significantly higher in the PCIA group than in the PCEA group before surgery (P < 0.01). The rate of occurrence of coagulation abnormalities on POD3 was significantly higher in the PCEA group than in the PCIA group (P < 0.001).

**Table 6:** Analgesia-related complication.

		PCEA	PCIA	P
**POD1**				
	PONV	190/858	199/849	0.6131
	Pruritus	219/829	41/1007	<.0001
	Hypotension	26/1022	4/1044	0.0002
	Respiratory depression	2/1046	0/1048	0.180
**POD2**				
	PONV	40/1008	49/999	0.3296
	Pruritus	84/964	18/1030	<.0001
	Hypotension	5/1045	0/1048	0.0289
	Respiratory depression	0/1048	1/1047	0.317
**Coagulation**				
	Preoperative coagulopathy	327/721	386/662	0.007
	Coagulopathy in POD3	870/178	804/244	0.0003
	Preoperative anticoagulants	20	45	0.002

A comparison of the intraoperative indicators of patients in Table 7 showed that colloid input amount and urine volume were significantly higher in the PCEA group than in the PCIA group, and the intraoperative blood loss and intraoperative opioid dosage were significantly lower in the PCEA group than in the PCIA group. The average postoperative hospital stay of patients in the PCEA group was one day shorter than that of patients in the PCIA group. Moreover, the incidence of postoperative surgical site infection and abnormal liver function was significantly higher in the PCEA group than in the PCIA group. The incidences of postoperative pulmonary complications, postoperative bleeding, and secondary surgery were significantly higher in the PCIA group than in the PCEA group. However, in similarity to the preoperative day, there was no statistically significant difference in serum creatinine on POD3 between patients in the PCEA and PCIA groups.

**Table 7:** Intraoperative fluid, opioid dosage and postoperative complications.

	PCEA	PCIA	P
Crystal solution(ml)	1349.2（595.6）	1399.1（630.9）	0.0639
Colloid Solution(ml)	466.7（275.7）	420.9（346.2）	0.0009
Blood loss(ml)	252.5（293.4）	296.0（401.1）	0.005
Urine volume(ml)	332.3（345.1）	272.8（257.8）	<.0001
Opioid dosage(mg)	29.290（3.740）	48.94（8.629）	<.0001
Hospital lengths of stay(days)	8.480（4.987）	9.412（4.783）	<.0001
Scr POD3(umol/L)	70.92(22.03)	71.32(28.98)	0.7622
Surgical site infections(n)	81/965	12/1036	<.0001
Pulmonary complications(n)	0/1046	10/1038	0.0015
Hepatic dysfunction or failure(n)	103/943	9/1039	<.0001
Massive bleeding(n)	5/1041	14/1034	0.0385
Exploratory laparotomy(n)	0/1046	8/1040	0.0046
Other complication(n)	6/1039	11/1037	0.2256

Scr POD3, serum creatinine on postoperative day 3. Data are expressed as mean (standard deviation) or number of cases (percentage).

## 4 Discussion

The present study compared the effectiveness of epidural analgesia and intravenous analgesia during postoperative analgesia and analgesia-related adverse effects after open hepatectomy. The effects of different analgesic modalities on patients’ postoperative motor function recovery, postoperative rehabilitation, and complications were investigated.

Our results showed that the NRS scores of the PCEA group were significantly lower than those of the PCIA group in all three states on POD1. On POD2, the NRS scores after exercise were lower in the PCEA group than in the PCIA group; however, the pain scores between the two groups in the other two conditions were not significantly different. The mean NRS scores in the three states of rest, exercise, and nocturnal pain were less than 3 in both groups within 2 days after surgery, thus indicating that both analgesic modalities provided good postoperative analgesia for most patients undergoing open hepatectomy. On POD2, no significant difference was observed between the two groups. The logistic regression analysis showed that the postoperative analgesic modality of PCEA was associated only with the incidence of moderate to severe pain in the resting and motor states on POD1, thus suggesting that PCEA is better than PCIA in relieving pain in motor states on POD1. Jesse V. Groen et al. reported a similar conclusion in their retrospective analysis of epidural analgesia and intravenous analgesia after open pancreatic surgery [8]. No significant difference between the two groups was observed in patient satisfaction with postoperative analgesia; this finding is consistent with that reported by Fayed et al. [9].

We also analyzed the effectiveness of patient self-administered medication for acute pain relief. On both POD1 and POD2, the effective compression ratio and the analgesic relief rate of patients in the PCEA group were significantly lower than those in the PCIA group. Operator-based technicalities are critical for the effectiveness of postoperative epidural analgesia. A study on the effectiveness of epidural analgesia in gastrointestinal surgery reported failure of epidural analgesia in up to 30 % of patients because of reasons such as improper placement of the catheter, catheter displacement, and poor drug dosing [10]. Moreover, the development of complications associated with perioperative epidural analgesia can lead to early discontinuation of the epidural analgesia pump, which in turn can affect the overall outcome of postoperative epidural analgesia. In a study of postoperative analgesia in patients undergoing open pancreatic surgery, an association was observed between postoperative epidural analgesia and perioperative hemodynamic instability, resulting in early termination of epidural analgesia in 10.5 % of patients [8]. In our present study, the incidence of postoperative hypotension and pruritus was significantly higher in the PCEA group than in the PCIA group, and the incidence of PONV and respiratory depression was similar in both groups; this finding is consistent with the discontinuation of analgesic pumps in both groups. In general, MAP<90 mmHg after abdominal surgery can be defined as postoperative hypotension [11]. Ward physicians usually dealt with discontinuation of analgesic pumps, observation, intensive monitoring, use of ephedrine, etc. If necessary, the surgeon will contact the postoperative acute pain management Team to adjust the background dose and bolus dose of the analgesic pump.

In this study, the FAS functional classification was used to evaluate the effect of pain on the motor and functional status of patients. On POD1, patients receiving postoperative epidural analgesia were less likely to have motor functional limitations due to pain as compared to those receiving postoperative intravenous analgesia. On POD2, the FAS functional class was again A in both groups, thus indicating that both analgesic modalities were effective in relieving pain and reducing the effect of pain on the motor function of patients on POD2. Epidural analgesia can effectively relieve motor pain on POD1 and provide good analgesia for performing early postoperative exercises in patients. Similar to our study, Bell et al. found that, except on POD1, epidural analgesia provided similar postoperative analgesic effects as those achieved with other analgesic modalities, and no difference was observed in postoperative outcomes of patients [12]. Jesse V. Groen et al. reported that PCEA had better analgesic effects than PCIA on POD1, and this difference disappeared on POD2; on POD3 and POD4, patients in the PCEA group experienced significantly more moderate to severe pain than those in the PCIA group. Moreover, after discontinuing epidural analgesia, the PCEA group showed increased mean pain scores and required remedial treatment with higher doses of opioids [8].

Advanced epidural catheterization technique is essential. Failure of the epidural catheter placement can lead to patients experiencing postoperative pain irritation, even severe pain irritation, thereby increasing the use of additional opioids for treatment. Therefore, successful epidural puncture placement, control of the associated factors to reduce analgesic pump discontinuation triggered by analgesia-related complications, and timely pain management for patients discontinuing epidural analgesia are very important in effectively relieving pain in patients after liver surgery. In a randomized controlled study of analgesia after hepatectomy, the researchers used a strict protocol for drug concentration, administered as a continuous dose and a bolus dose, with a lower than commonly reported concentration of 0.075 % bupivacaine as a local anesthetic; they achieved good analgesic effects while avoiding hypotension and other complications associated with epidural analgesia [1]. Postoperative multimodal analgesia protocols should be effective in improving the efficiency and relief rates of self-administered postoperative epidural analgesia; however, further randomized controlled studies with large sample sizes are required to determine the appropriate protocols for pain relief in postoperative patients.

In our present study, approximately 35.5 % of patients who underwent postoperative PCEA had preoperative coagulation dysfunction. In our center, the epidural catheter was generally removed on POD3, and on the day of removal, coagulation abnormalities occurred in approximately 83.02 % of patients in the epidural analgesia group. For the patients with coagulation abnormality, the anesthesiologist decided whether to perform the epidural catheterization according to their own judgement and risk versus benefit. Coagulation is mildly abnormal in patients undergoing epidural catheterization. Also, the anesthesiologist will remove the epidural catheter when the patient's postoperative coagulation is relatively normal. None of the patients developed an epidural hematoma in present research.

In this study, we used the definition of coagulation abnormalities as meeting any of the following three criteria: INR >1.5; APTT >40 s; PLT count <80 × 10^9^/L [7]. In a study on coagulation during postoperative epidural catheter withdrawal in patients undergoing hepatectomy, the changes were most severe on POD2 and persisted until at least POD5–POD6 [13,14]. However, this may not reflect the actual coagulation status of the patients. Some studies have investigated the anticoagulation system and found that patients were in a hypercoagulable state in the early postoperative period [5]. Ejaz et al. reported a higher incidence of venous thromboembolism after hepatectomy than after most other major abdominal procedures [15]. In another study on the changes in the coagulation status of patients during epidural catheter removal, it was suggested that only 34 % of patients with coagulation abnormalities received fresh frozen plasma during epidural catheter removal, while INR ≥1.7 (the “unsafe” criterion) was noted n 23 % of patients. Moreover, none of the patients with “accidental epidural catheter dislodgement” (10/121 [8.3 %]) developed an epidural hematoma [13]. Mallett et al. studied coagulation abnormalities in patients undergoing hepatectomy and found that thromboelastography in patients showed normal results throughout the procedure [16]. This finding suggests that the balance of pro- and anticoagulant systems reduces both pro- and anticoagulant factors, and that elevated levels of Factor VIII and von Willebrand factor (VWF) favor thrombosis, rather than an increase in bleeding risk. Therefore, a more comprehensive assessment of coagulation abnormalities is required to evaluate whether epidural analgesia can be safely used in patients undergoing hepatectomy and to determine the timing of safe removal of the epidural catheter. In patients with mild preoperative coagulation abnormalities, the risk–benefit ratio of epidural analgesia administration should be evaluated to make an informed decision regarding its use during surgery.

In line with previous studies, postoperative epidural analgesia reduced the amounts of intraoperative opioids used by patients and the incidence of postoperative-related complications in patients, shortened the length of hospital stay, and facilitated the recovery of patients [17]. In our present study, however, we found that the incidence of postoperative liver function abnormalities was significantly higher in the PCEA group than in the PCIA group; this was probably because the diagnosis of complications was included in the surgical records and was related to the clinical criteria for the diagnosis of liver function abnormalities. In our clinical practice, anesthesiologists tend to prefer the use of epidural analgesia for patients with a larger tumor diameter or for patients requiring more resection of liver tissue, and the increased resection margin may lead to an increased incidence of abnormal liver function. The present study also found that intraoperative colloid infusion was significantly higher in the PCEA group than in the PCIA group, while no significant difference was observed in the amount of crystalloid infusion. Epidural analgesia also reduced intraoperative bleeding in patients, which may be related to dilation of the peripheral vasculature by the epidural block and reduction in blood flow to the surgical area.

Postoperative infections are an important health challenge in wards. With regard to treatment, modifiable factors such as source control and antimicrobial therapy play a key role in influencing the prognosis of postoperative infections, which up to the surgeon to make the decision [18]. Interestingly, the incidence of postoperative surgical site infection was significantly higher in the PCEA group than in the PCIA group. We retraced the reasons that led to infections in the patients in this study, which may be related to surgical procedures, the patients' own obesity, malnutrition, and debilitated state. Of course, an increased rate of postoperative infections cannot be excluded in relation to the choice of epidural anesthesia. However, retrospective studies may be potentially biased, and the relationship between postoperative infections and epidural anesthesia requires further study. Acute kidney injury is a frequent postoperative complication that carries a substantial risk for postoperative adverse outcomes, as well as long-term mortality and morbidity. To prevent postoperative AKI, avoiding hypotension and hypoperfusion are important [19]. The present study showed there was no statistically significant difference in serum creatinine on POD3 between patients in the PCEA and PCIA groups. This phenomenon may be based on the mild degree of postoperative hypotension in patients, which was promptly and effectively managed.

The advantages of our study are that instead of simple comparisons between two pain management options, we used Propensity Score Matching (PSM) to balance baseline information to minimize statistical bias from retrospective studies. This study may also provide some decision-making basis for clinical anesthesiologists on postoperative pain management in liver surgery. The present study was a single-center study, which restricts to some extent the external validity of our findings. Moreover, because of the retrospective nature of the study, data collection may have human errors in the interpretation and recording of data. Electronic medical records are widely used in our institution, and data collection was performed by investigators who were not involved in intraoperative and postoperative management; moreover, the collected data were validated by two other investigators to minimize the possibility of detection bias. Retrospective studies are not randomized or blinded, and there may be individual differences in recommending epidural analgesia in clinical practice; additionally, the decision to use epidural anesthesia is related to the preference of the anesthetist, the patient's general condition, and the surgical plan. Nevertheless, because of the large sample size of our present study, the findings can serve as a reference for future clinical practice. In addition, in our study, analgesic follow-up was performed only on two days: POD1 and POD2. Thus, changes in patient pain scores, occurrence of flare-ups, and pharmacological remediation after discontinuation of epidural or intravenous analgesia remain unknown; hence, prospective, randomized controlled trials are required to investigate multimodal postoperative analgesia protocols for improving the effectiveness of epidural analgesia.

## 5 Conclusion

In patients undergoing open hepatectomy, both epidural analgesia and intravenous analgesia can provide good analgesic effects, and epidural analgesia can be effective in relieving the onset of moderate to severe pain on the first postoperative day at rest and during motor activity. Additional investigations are required to improve the puncture success rate, reduce analgesia-related adverse reactions that lead to analgesic pump discontinuation, and include multimodal analgesia after the removal of the epidural catheter to further improve the effectiveness of epidural analgesia. The coagulation status of the patient should be thoroughly evaluated when selecting epidural analgesia to improve the safety of epidural use in patients undergoing open hepatectomy.

## Funding

This article was supported by the Research Funds of Zhongshan Hospital, Fudan University (2021ZSQN45).

## Data availability statement

Data will be made available on request.

## CRediT authorship contribution statement

**Xue-Peng Zhang:** Writing – original draft, Methodology, Investigation, Formal analysis, Conceptualization. **Wan-Ting Wei:** Software, Investigation, Formal analysis. **Yong Huang:** Software. **Chang-Hong Miao:** Supervision. **Xiao-Guang Zhang:** Writing – review & editing, Supervision, Data curation. **Fang Du:** Writing – review & editing, Validation, Supervision, Project administration, Funding acquisition, Conceptualization.

## Declaration of competing interest

The authors declare that they have no known competing financial interests or personal relationships that could have appeared to influence the work reported in this paper.
