# Supplementary material for: Efficacy and safety of patient-controlled epidural analgesia versus patient-controlled intravenous analgesia following open hepatectomy: A single-center retrospective study
Source: Heliyon. 2023 Dec 14;10(1):e23548. doi: 10.1016/j.heliyon.2023.e23548 (PMC10767150; doi:10.1016/j.heliyon.2023.e23548)
Supplement: Multimedia component 3 [file mmc3.docx]

3、NRS Logistics regression analysis：

POD1：

1. rest：

|  | B Value | standard error | degrees of freedom | Wald  chi-square | P | OR | 95% CI | |
| --- | --- | --- | --- | --- | --- | --- | --- | --- |
| constant | 7.0484 | 343.4 | 1 | 0.0004 | 0.9836 |  |  |  |
| age | -0.0345 | 0.0442 | 1 | 0.6085 | 0.4353 | 0.966 | 0.886 | 1.053 |
| BMI | 0.0290 | 0.1677 | 1 | 0.0300 | 0.8625 | 1.029 | 0.741 | 1.430 |
| clusters | -1.2701 | 1.2288 | 1 | 1.0683 | 0.3013 | 0.281 | 0.025 | 3.122 |
| genders | -0.5945 | 1.4274 | 1 | 0.1735 | 0.6770 | 0.552 | 0.034 | 9.054 |
| c1 ASA | 0.5851 | 1.0971 | 1 | 0.2844 | 0.5938 | 1.795 | 0.209 | 15.415 |
| c2 Child | -5.2626 | 308.0 | 1 | 0.0003 | 0.9864 | 0.005 | <0.001 | >999.999 |
| C3 ALBI | -11.3145 | 154.8 | 1 | 0.0053 | 0.9417 | <0.001 | <0.001 | >999.999 |
| C4 secondary operation | 1.1429 | 1.1080 | 1 | 1.0641 | 0.3023 | 3.136 | 0.357 | 27.508 |
| C5 surgery duration | 0.00692 | 0.00403 | 1 | 2.9441 | 0.0862 | 1.007 | 0.999 | 1.015 |
| E1surgeon | 0.0476 | 0.0619 | 1 | 0.5895 | 0.4426 | 1.049 | 0.929 | 1.184 |
| E2anesthesi-  ologist | -0.0376 | 0.0430 | 1 | 0.7649 | 0.3818 | 0.963 | 0.885 | 1.048 |
| E3 incision | -10.2951 | 253.0 | 1 | 0.0017 | 0.9675 | <0.001 | <0.001 | >999.999 |
| E4 type of surgery | 1.4083 | 0.5399 | 1 | 6.8035 | 0.0091 | 4.089 | 1.419 | 11.782 |
| E5tumor size | 0.8236 | 0.7437 | 1 | 1.2267 | 0.2681 | 2.279 | 0.531 | 9.788 |
| E6 number of pringle maneuver | -0.4131 | 0.9457 | 1 | 0.1908 | 0.6623 | 0.662 | 0.104 | 4.223 |
| E7 time of pringle maneuver | 0.0587 | 0.0551 | 1 | 1.1354 | 0.2866 | 1.060 | 0.952 | 1.181 |

1. motion

|  | B Value | standard error | degrees of freedom | Wald  chi-square | P | OR | 95% CI | |
| --- | --- | --- | --- | --- | --- | --- | --- | --- |
| constant | -0.3661 | 1.5741 | 1 | 0.0541 | 0.8161 |  |  |  |
| age | -0.0279 | 0.00932 | 1 | 8.9825 | 0.0027 | 0.972 | 0.955 | 0.990 |
| BMI | -0.0209 | 0.0348 | 1 | 0.3582 | 0.5495 | 0.979 | 0.915 | 1.049 |
| clusters | -0.8081 | 0.2386 | 1 | 11.4734 | 0.0007 | 0.446 | 0.279 | 0.711 |
| genders | 0.0271 | 0.2522 | 1 | 0.0115 | 0.9145 | 1.027 | 0.627 | 1.684 |
| c1ASA | 0.0957 | 0.2280 | 1 | 0.1761 | 0.6748 | 1.100 | 0.704 | 1.721 |
| c2Child | -0.7841 | 1.0600 | 1 | 0.5473 | 0.4594 | 0.457 | 0.057 | 3.645 |
| C3ALBI | -0.3835 | 0.2899 | 1 | 1.7495 | 0.1859 | 0.681 | 0.386 | 1.203 |
| C4 secondary operation | 0.1457 | 0.2805 | 1 | 0.2698 | 0.6035 | 1.157 | 0.668 | 2.005 |
| C5 surgery duration | 0.00214 | 0.00136 | 1 | 2.4829 | 0.1151 | 1.002 | 0.999 | 1.005 |
| E1 surgeon | -0.0188 | 0.0138 | 1 | 1.8488 | 0.1739 | 0.981 | 0.955 | 1.008 |
| E2anesthesi-ologist | 0.0176 | 0.0101 | 1 | 3.0227 | 0.0821 | 1.018 | 0.998 | 1.038 |
| E3 incision | 0.1721 | 0.2155 | 1 | 0.6379 | 0.4245 | 1.188 | 0.779 | 1.812 |
| E4 type of surgery | 0.1504 | 0.1544 | 1 | 0.9491 | 0.3300 | 1.162 | 0.859 | 1.573 |
| E5 tumor size | 0.0441 | 0.1777 | 1 | 0.0615 | 0.8042 | 1.045 | 0.738 | 1.480 |
| E6 number of pringle maneuver | -0.1301 | 0.2124 | 1 | 0.3754 | 0.5401 | 0.878 | 0.579 | 1.331 |
| E7 time of pringle maneuver | 0.0149 | 0.0125 | 1 | 1.4206 | 0.2333 | 1.015 | 0.990 | 1.040 |

1. night

|  | B Value | standard error | degrees of freedom | Wald  chi-square | P | OR | 95% CI | |
| --- | --- | --- | --- | --- | --- | --- | --- | --- |
| constant | -1.7463 | 2.3254 | 1 | 0.5640 | 0.4527 |  |  |  |
| age | -0.0321 | 0.0158 | 1 | 4.1241 | 0.0423 | 0.968 | 0.939 | 0.999 |
| BMI | -0.0935 | 0.0639 | 1 | 2.1411 | 0.1434 | 0.911 | 0.804 | 1.032 |
| clusters | -0.2215 | 0.4732 | 1 | 0.2191 | 0.6397 | 0.801 | 0.317 | 2.026 |
| genders | 0.2760 | 0.4171 | 1 | 0.4378 | 0.5082 | 1.318 | 0.582 | 2.984 |
| c1ASA | 0.6169 | 0.4199 | 1 | 2.1583 | 0.1418 | 1.853 | 0.814 | 4.220 |
| c2Child | 1.7183 | 1.2065 | 1 | 2.0285 | 0.1544 | 5.575 | 0.524 | 59.324 |
| C3ALBI | -1.3441 | 0.7201 | 1 | 3.4844 | 0.0619 | 0.261 | 0.064 | 1.069 |
| C4 secondary operation | -0.2639 | 0.5672 | 1 | 0.2165 | 0.6417 | 0.768 | 0.253 | 2.334 |
| C5 surgery duration | -0.00169 | 0.00285 | 1 | 0.3537 | 0.5520 | 0.998 | 0.993 | 1.004 |
| E1 surgeon | 0.00198 | 0.0242 | 1 | 0.0066 | 0.9350 | 1.002 | 0.955 | 1.051 |
| E2anesthesi-ologist | -0.00720 | 0.0168 | 1 | 0.1825 | 0.6693 | 0.993 | 0.961 | 1.026 |
| E3 incision | -0.0646 | 0.4597 | 1 | 0.0197 | 0.8883 | 0.937 | 0.381 | 2.308 |
| E4 type of surgery | 0.1412 | 0.2766 | 1 | 0.2607 | 0.6096 | 1.152 | 0.670 | 1.980 |
| E5 tumor size | 0.5690 | 0.3128 | 1 | 3.3077 | 0.0690 | 1.766 | 0.957 | 3.261 |
| E6 number of pringle maneuver | -0.4835 | 0.4009 | 1 | 1.4544 | 0.2278 | 0.617 | 0.281 | 1.353 |
| E7 time of pringle maneuver | 0.000185 | 0.0216 | 1 | 0.0001 | 0.9931 | 1.000 | 0.959 | 1.043 |

POD2：

1. rest
2. motion

|  | B Value | standard error | degrees of freedom | Wald  chi-square | P | OR | 95% CI | |
| --- | --- | --- | --- | --- | --- | --- | --- | --- |
| constant | -9.1975 | 3.4169 | 1 | 7.2455 | 0.0071 |  |  |  |
| age | -0.0497 | 0.0243 | 1 | 4.1734 | 0.0411 | 0.952 | 0.907 | 0.998 |
| BMI | 0.1298 | 0.0812 | 1 | 2.5541 | 0.1100 | 1.139 | 0.971 | 1.335 |
| clusters | -0.7186 | 0.5977 | 1 | 1.4452 | 0.2293 | 0.487 | 0.151 | 1.573 |
| genders | 0.1245 | 0.6823 | 1 | 0.0333 | 0.8552 | 1.133 | 0.297 | 4.314 |
| c1ASA | 0.2896 | 0.6327 | 1 | 0.2096 | 0.6471 | 1.336 | 0.387 | 4.617 |
| c2Child | 2.9579 | 1.3793 | 1 | 4.5989 | 0.0320 | 19.258 | 1.290 | 287.529 |
| C3ALBI | -0.9088 | 0.9548 | 1 | 0.9060 | 0.3412 | 0.403 | 0.062 | 2.618 |
| C4 secondary operation | -11.2037 | 185.3 | 1 | 0.0037 | 0.9518 | <0.001 | <0.001 | >999.999 |
| C5 surgery duration | -0.00725 | 0.00488 | 1 | 2.2124 | 0.1369 | 0.993 | 0.983 | 1.002 |
| E1 surgeon | 0.0805 | 0.0393 | 1 | 4.2036 | 0.0403 | 1.084 | 1.004 | 1.171 |
| E2anesthesi-ologist | 0.0263 | 0.0258 | 1 | 1.0353 | 0.3089 | 1.027 | 0.976 | 1.080 |
| E3 incision | -9.9859 | 179.6 | 1 | 0.0031 | 0.9557 | <0.001 | <0.001 | >999.999 |
| E4 type of surgery | -0.1609 | 0.7322 | 1 | 0.0483 | 0.8261 | 0.851 | 0.203 | 3.576 |
| E5 tumor size | 0.1190 | 0.4458 | 1 | 0.0713 | 0.7894 | 1.126 | 0.470 | 2.699 |
| E6 number of pringle maneuver | 0.0548 | 0.5018 | 1 | 0.0119 | 0.9130 | 1.056 | 0.395 | 2.825 |
| E7 time of pringle maneuver | 0.0466 | 0.0263 | 1 | 3.1401 | 0.0764 | 1.048 | 0.995 | 1.103 |

1. night

|  | B Value | standard error | degrees of freedom | Wald  chi-square | P | OR | 95% CI | |
| --- | --- | --- | --- | --- | --- | --- | --- | --- |
| constant | 8.9057 | 494.7 | 1 | 0.0003 | 0.9856 |  |  |  |
| age | 0.00298 | 0.0221 | 1 | 0.0182 | 0.8925 | 1.003 | 0.961 | 1.047 |
| BMI | 0.0333 | 0.0759 | 1 | 0.1930 | 0.6605 | 1.034 | 0.891 | 1.200 |
| clusters | -0.3963 | 0.4964 | 1 | 0.6373 | 0.4247 | 0.673 | 0.254 | 1.780 |
| genders | -0.5914 | 0.6455 | 1 | 0.8393 | 0.3596 | 0.554 | 0.156 | 1.962 |
| c1ASA | -0.8838 | 0.4852 | 1 | 3.3179 | 0.0685 | 0.413 | 0.160 | 1.069 |
| c2Child | -10.9160 | 494.7 | 1 | 0.0005 | 0.9824 | <0.001 | <0.001 | >999.999 |
| C3ALBI | -0.7618 | 0.7804 | 1 | 0.9529 | 0.3290 | 0.467 | 0.101 | 2.155 |
| C4 secondary operation | -12.1971 | 204.8 | 1 | 0.0035 | 0.9525 | <0.001 | <0.001 | >999.999 |
| C5 surgery duration | -0.00146 | 0.00391 | 1 | 0.1385 | 0.7098 | 0.999 | 0.991 | 1.006 |
| E1 surgeon | 0.0180 | 0.0307 | 1 | 0.3436 | 0.5577 | 1.018 | 0.959 | 1.081 |
| E2anesthesi-ologist | -0.00159 | 0.0219 | 1 | 0.0053 | 0.9418 | 0.998 | 0.957 | 1.042 |
| E3 incision | -10.2286 | 216.2 | 1 | 0.0022 | 0.9623 | <0.001 | <0.001 | >999.999 |
| E4 type of surgery | 0.2021 | 0.3520 | 1 | 0.3298 | 0.5658 | 1.224 | 0.614 | 2.440 |
| E5 tumor size | -0.5342 | 0.4733 | 1 | 1.2740 | 0.2590 | 0.586 | 0.232 | 1.482 |
| E6 number of pringle maneuver | 0.1553 | 0.4998 | 1 | 0.0965 | 0.7560 | 1.168 | 0.439 | 3.111 |
| E7 time of pringle maneuver | 0.0139 | 0.0268 | 1 | 0.2681 | 0.6046 | 1.014 | 0.962 | 1.069 |
